# Quantitative Proteomic Profiling the Molecular Signatures of Annexin A5 in Lung Squamous Carcinoma Cells

**DOI:** 10.1371/journal.pone.0163622

**Published:** 2016-09-29

**Authors:** Bing Sun, Yuxin Bai, Liyuan Zhang, Linlin Gong, Xiaoyu Qi, Huizhen Li, Faming Wang, Xinming Chi, Yulin Jiang, Shujuan Shao

**Affiliations:** 1 Department of Thoracic Surgery, The First Affiliated Hospital of Dalian Medical University, Dalian, 116011, Liaoning, China; 2 Key Laboratory for Proteomics of Liaoning Province, Dalian Medical University, Lvshun South Road No 9, Dalian, 116044, Liaoning, China; University of Edinburgh, UNITED KINGDOM

## Abstract

Lung cancer remains the leading cancer killer around the world. It’s crucial to identify newer mechanism-based targets to effectively manage lung cancer. Annexin A5 (ANXA5) is a protein kinase C inhibitory protein and calcium dependent phospholipid-binding protein, which may act as an endogenous regulator of various pathophysiological processes. However, its molecular mechanism in lung cancer remains poorly understood. This study was designed to determine the mechanism of ANXA5 in lung cancer with a hope to obtain useful information to provide a new therapeutic target. We used a stable isotope dimethyl labeling based quantitative proteomic method to identify differentially expressed proteins in NSCLC cell lines after ANXA5 transfection. Out of 314 proteins, we identified 26 and 44 proteins that were down- and up-regulated upon ANXA5 modulation, respectively. The IPA analysis revealed that glycolysis and gluconeogenesis were the predominant pathways modulated by ANXA5. Multiple central nodes, namely HSPA5, FN1, PDIA6, ENO1, ALDOA, JUP and KRT6A appeared to occupy regulatory nodes in the protein-protein networks upon ANXA5 modulation. Taken together, ANXA5 appears to have pleotropic effects, as it modulates multiple key signaling pathways, supporting the potential usefulness of ANXA5 as a potential target in lung cancer. This study might provide a new insight into the mechanism of ANXA5 in lung cancer.

## 1. Introduction

Lung cancer is the leading cancer killer around the world [1], over 85% of lung cancers are diagnosed as non-small cell lung cancer (NSCLC). Even though major progress in the understanding of cancer biology and treatment of lung cancer has been achieved over the last few years, the 1-year survival rate for NSCLC patients is still disappointing, which is below 15%[2]. More research is urgently needed to explore new mechanism-based modalities in oncogenesis and cancer progression.

Annexin A5 (ANXA 5) is a non-glycosylated phospholipid binding protein composed of 319 amino acid residues with a molecular mass of ~35.7 kDa [3–5], which could bind to negatively charged phospholipids in a Ca^2+^-dependent manner. Compared with other annexins, ANXA5 has a very short unphosphorylated N-terminus. ANXA5 has been attributed many functions, including an involvement in cell proliferation and invasion [6–8], signal transduction [9, 10], and anticoagulation[11, 12]. In addition, ANXA5 was found to be associated with hepatocarcinoma[13, 14], cervical carcinoma[15, 16], colorectal cancer[17, 18] and other cancers. ANXA5 also has been reported to inhibit diffuse large B-cell lymphoma cell invasion and chemo-resistance [9], and the expression of ANXA5 may induce mitochondrial apoptosis in prostate cancer cells [19]. However, over expression of ANXA5 significantly increases cell invasion and promotes chemo-resistance to temozolomide in glioblastoma multiforme cells[20].

In our previous report [6, 21], ANXA5 was found to be down-regulated in lung squamous cell carcinoma (LUSC), compared with adjacent normal tissues, which is consistent with our result conducted from The Cancer Genome Atlas (TCGA). We have demonstrated that upregulation of ANXA5 inhibits the proliferation, migration, and invasion abilities of NCI-H520 cells in vitro. We have also demonstrated that upregulation of ANXA5 decreases the expression of vimentin and increases the expression of E-cadherin. However, the precise biological role of ANXA5 in the living lung cancer cell is still far from clear.

Quantitative proteomic techniques have recently emerged as a powerful approach to uncover the differential proteins expression associated with mechanism and signal pathway, with a high-degree of specificity and sensitivity. Nowadays, stable isotope dimethyl labeling based quantitative proteomic approach is one of the most popular techniques for quantitative proteomic analysis, with the advantages of universality, fast and high derivatization efficiency[22–24]. In this study, the molecular mechanism of ANXA5 in NSCLC was deciphered by interrogating the proteomics changes after ANXA 5-transfecting. As a result, 70 differentially expressed proteins (from a total of 314) were identified, which may be useful in creating a molecular signature of ANXA5 in lung cancer.

## 2. Materials and Methods

### 2.1 Chemicals reagents and antibodies

RPMI 1640 and fetal bovine serum (FBS) were obtained from HyClone Laboratories (HyClone Laboratories Inc.). Dimethyl-labeled agents, CH_2_O, CD_2_O, NaCNBH_3_ were obtained from Sigma-Aldrich Company (St. Louis, MO). The primary antibodies for Hsp90 alpha, ENO1, ALDOA, HSPA5, JUP, KRT6A, PDIA6, AKT, PCNA, FN1, β-actin and all the secondary antibodies were obtained from Proteintech Group (Proteintech Group, Inc., USA).

All other agents were purchased from Sigma Chemical Co. (St. Louis, MO) unless otherwise specified. The water used in the experiments was thrice-distilled; all other materials were of analytical reagent grade.

### 2.2 Cell culture, gene transfection, and screening

NCI-H520 cells were routinely cultured in RPMI1640 media supplemented with 10% fetal bovine serum (FBS) at 37°C under humidified atmosphere containing 5% CO_2_. The cells were passaged with 0.25% trypsin. The pCDNA3.1-ANXA5 and control plasmids were transfected with Lipofectamine 2000 (Invitrogen, CA, USA) into NCI-H520 cells following the manufacturer’s instructions. The tested cells were divided into three groups: pCDNA3.1-NCI-H520-ANXA5 transfected cells, pCDNA3.1-NCI-H520-NC and NCI-H520. After being screened by 800 μg/mL G418 (Sigma, MO, USA) for 14 days, the cells were then continuously cultured in a regular medium containing 400 μg/mL G418.

### 2.3 Protein Preparation and Tryptic Digestion

The cells of pCDNA3.1-NCI-H520-ANXA5, pCDNA3.1-NCI-H520-NC and NCI-H520 were harvested for protein extraction on ice. The cell pellets were dissolved in a cell lysis buffer (8 M urea) plus 1% (v/v) protease inhibitor cocktail set. The suspension was homogenized on ice for 1 min, ultrasonicated for 30 s and centrifuged at 25000 g for 30 min at 4°C, and the supernatants were harvested and frozen at -80°C. Protein concentrations were determined using a BCA protein assay kit according to the manufacturer’s instructions. Proteins were reduced with 10 mM dithiothreitol for 30 min at 56°C, then alkylated with 20 mM iodoacetamide for 1 h at room temperature in the dark. Cell lysates were diluted and digested with a trypsin to protein ratio of 1:25 (W/W) at 37°C overnight. The resulting peptide solutions were acidified with 1% FA, and desalted on a C18 trap column. Eluted peptides were lyophilized to complete dryness, and stored at -80°C until needed.

### 2.4 Stable isotope dimethyl labeling

Stable isotope dimethyl labeling was performed according to the reported protocol [22] with the appropriate improvements. Briefly, the proteins extracted from NCI-H520 and pCDNA3.1-NCI-H520-ANXA5 cells were labeled by light (0.2% CH_2_O and 30 mM NaBH_3_CN) and heavy (0.2% CD_2_O and 30 mM NaBH_3_CN) dimethylation reagents, respectively. After keeping the reaction solution in 25°C for 1 h, 2 mL of 10% (vol/vol) ammonia and 5 mL of 10% (vol/vol) formic acid in water were successively added to quench the reaction. The isotopically labeled peptides were mixed together, desalted by a C18 solid-phase extraction column, lyophilized to powder and re-dissolved in 0.1% FA in H_2_O for the following LC-MS/MS analysis.

### 2.5 NanoLC−MS/MS Analysis

The peptide samples were analyzed by nano-RPLC-ESI-MS/MS with an LTQ-Orbitrap Elite mass spectrometer (Thermo Fisher Scientific, San Jose, CA, USA) equipped with a Dionex ultimate 3000 liquid chromatography and an ESI probe Ion Max Source with a nanospray kit. The spectrometer was controlled by Xcalibur software version 2.2 (Thermo Fisher, Waltham, MA, USA). The peptides were separated on a C18 capillary column (30 cm, 75 μ m i.d./375 μ m o.d.) packed with C18 silica particles (5 μ m, 100 Å) with a 145 min gradient from 10 to 40% buffer B (98% ACN/0.1% FA) and analyzed on the mass spectrometer. Mass spectra were acquired in a data-dependent mode. MS1 spectra were measured at a resolution of 6 × 10^4^ and the top 10 most abundant ions with an isolation window of 2 m/z were selected for sequencing and fragmented in the data-dependent CID mode with a normalized collision energy of 35%, activation Q of 0.25, activation time of 10 ms, and one microscan. The sample was analyzed in quadruplicate. Raw mass spectrometric data files can be downloaded from ftp://massive.ucsd.edu/MSV000079911. The evidence table containing protein data is provided as S1 Table.

### 2.6 Data Processing

Data analysis was accomplished by using MaxQuant software (http://maxquant.org/, version 1.3.0.3) against Uniprot human database (201504). Peptides were searched using the following parameters: fully tryptic cleavage constraints; up to two internal cleavage sites allowed for tryptic digestion; carbamidomethylation as a fixed modification; oxidation of methionine and protein N-terminal acetylation as variable modifications; dimethyl (+ 28.0313 Da) and dimethyl (+ 32.0564) N-termini and K set as light/heavy labels for quantification. The peptide mass tolerance was set at 20 ppm and MS/MS tolerance was set at 0.5 Da. Protein and peptides FDRs were 1%. The rest of the parameters follow the default settings of MaxQuant software.

### 2.7 Bioinformatics Analysis

The gene ontology annotations including cellular component, biological process, and molecular function were performed using DAVID (http://david.abcc.ncifcrf.gov)[25, 26].

The canonical pathways and protein-protein interactions were analyzed using Ingenuity Pathway Analysis Software (http://www.ingenuity.com)[27]. The predicted protein-protein interaction networks and canonical pathways were generated using inputs of gene identifiers, log2 fold-changes and p-values between pCDNA3.1-NCI-H520-ANXA5 and NCl-H520 group comparisons.

### 2.8 Western blot analysis

The cells of pCDNA3.1-NCI-H520-ANXA5, pCDNA3.1-NCI-H520-NC and NCl-H520 were collected and lysed in cell lysis buffer for Western (Beyotime Biotechnology, Shanghai, China). The concentration of proteins was determined using a BCA protein assay kit. Cell lysate proteins (40 μg) were separated by electrophoresis on a sodium dodecyl sulfate-polyacrylamide minigels (SDS-PAGE) gel and then electrophoretically transferred to PVDF membranes. After that, the membranes were incubated with 5% dehydrated skim milk for 2 h at room temperature. Western blots were probed with the specific antibodies. Proteins were detected by enhanced chemiluminescence system according to the manufacturer’s instructions. Similar experiments were performed at least three times.

### 2.9 Statistical analysis

All experiments were repeated at least three times. Analysis of variance (ANOVA) and Fisher's Least Significant Difference (LSD) test were used to compare the values of the test and control samples. P<0.05 was considered to be a statistically significant difference. SPSS 19.0 software was used for all statistical analysis.

### 2.10 TCGA analysis method

A cohort of 178 samples of lung squamous cell carcinoma (LUSC) taken from primary tumor specimen was downloaded from the TCGA database in December 01, 2015 based on availability of both mutations, mRNA expressions and copy number data [28]. A cohort of 230 samples of lung adenocarcinoma (LUAD) taken from primary tumor specimen was downloaded from the TCGA database in December 01, 2015 based on availability of both mutations, mRNA expressions and copy number data[29]. The copy number alterations were derived from GISTIC 2.0 [30] and all analysis was performed using cBioPortal [31].

## 3. Results

### 3.1 ANXA5 is down-regulated in NSCLC patients

According to the results from TCGA, the expression levels of ANXA5 in NSCLC tissues were significantly lower in lung squamous cell carcinoma (LUSC) and lung adenocarcinoma (LUAD) (Table 1) compared to matched normal samples, which is consistent with our proteomic result from LUSC and adjacent normal tissues [6, 21].

**Table 1:** The expression level of ANXA5 in LUSC and LUAD patients.

Cancer Type	Log(Fold-Change)	p-Value	FDR
LUSC	-0.91883	1.12×10^−26^	6.41×10^−26^
LUAD	-0.3848	3.17×.1^−07^	7.48×.4^−07^

Besides, ANXA5 was altered in 3 (1.7%, all 178 cases) from TCGA [28] in LUSC, in which two of them are missense mutations and the another one is truncating mutation. The mutation frequency of ANXA5 in LUSC is very low. For copy number alterations, there are 70 shallow deletions (39.3%), 101 diploids (56.7%) and 7 gains (3.9%) in 178 LUSC patients from TCGA [28]. For frequent loss, there are 70 (39.3%) shallow deletions in 178 LUSC patients.

### 3.2 Proteome profile of the effect of ANXA5 on NCl-H520 cells

Our early study had shown that there was no difference between NCI-H520 and pCDNA3.1-NCI-H520-NC cells (P<0.05) in cell proliferation, migration and invasion [6], according to results from the morphological analysis by crystal violet staining, the proliferation analysis by CCK8 and colony formation assays, indicating that the stress of transfection had no significant impact on the phenotype of the cells and their proteome. To elucidate the effect of ANXA5 on global proteome changes in NCl-H520 cells, the total cell lysates of ANXA5-transfected and wide type NCl-H520 cells were extracted. Stable isotope dimethyl labeling based quantitative proteomics strategy was applied for differential protein discovery.

**Fig 1:**
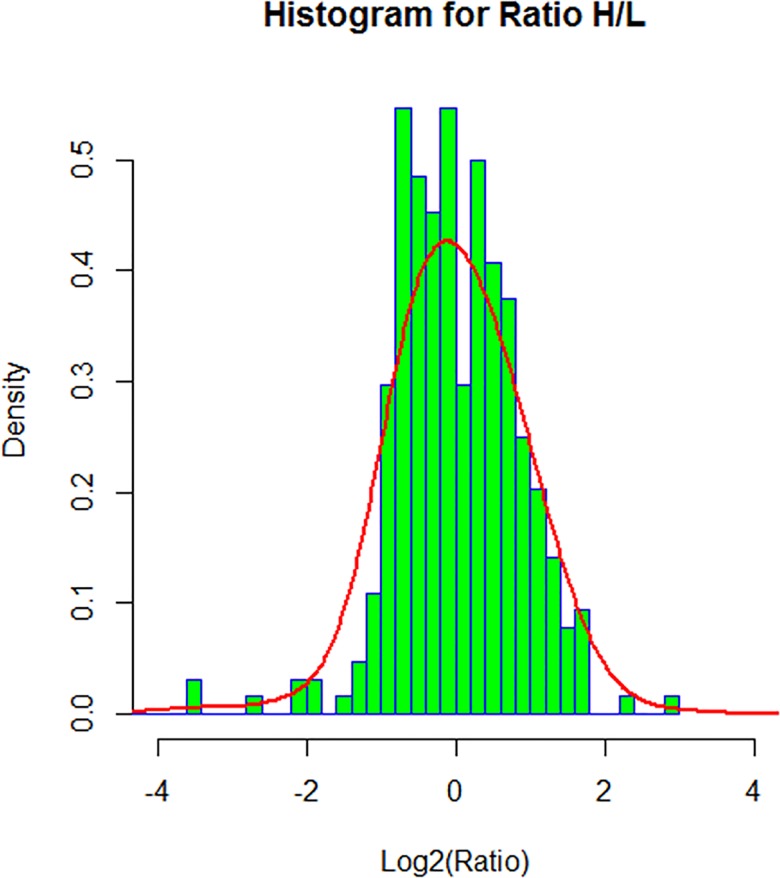
Histogram representation of protein abundance changes upon ANXA5 modulation.

In order to select the significant difference expressed proteins with a sound statistics base, we analyzed the global distribution of the Ratio H/L (normalized) calculated by MaxQuant software. A normal distribution fitting was applied to the log_2_(Ratio H/L). The mean (μ) and standard division (σ) of the normal distribution was used to determine the significant difference range, which is log_2_(Ratio H/L)>μ+σ or log_2_(Ratio H/L)<μ-σ, as shown in the histogram plot of Fig 1. As a result, 314 proteins were quantified, of which, 70 proteins (44 upregulated and 26 downregulated) showed different expression. Details about these 70 proteins, including protein ID, protein name, gene name and fold change regulated by ANXA5, are presented in Table 2. It is worth noting that, among these identified proteins, five proteins (Hsp90, ENO1, ALDOA, PDIA6, HSPA5) were reported as the differential proteins identified in lung cancer tissues as compared to normal lung in our previous work [21].

**Table 2:** The list of differentially expressed proteins in H520 cells modulated by ANXA5.

Protein ID	Protein Names	Gene Name	Log-transformed Ratio
P09429	High mobility group protein B1	HMGB1	1.7650
O00299	Chloride intracellular channel protein 1	CLIC1	1.1982
O00592	Podocalyxin	PODXL	-1.1293
O14684	Prostaglandin E synthase	PTGES	0.9313
O43707	Alpha-actinin-4	ACTN4	0.8955
O95292	Vesicle-associated membrane protein-associated protein B/C	VAPB	-0.8758
O95399	Urotensin-2	UTS2	-1.8955
P00338	L-lactate dehydrogenase A chain	LDHA	1.6220
P00367	Glutamate dehydrogenase 1, mitochondrial	GLUD1	-0.9197
P00558	Phosphoglycerate kinase 1	PGK1	1.2099
P02533	Keratin, type I cytoskeletal 14	KRT14	-3.4349
P02538	Keratin, type II cytoskeletal 6A	KRT6A	-2.6446
P02751	Fibronectin	FN1	2.2461
P04075	Fructose-bisphosphatealdolase A	ALDOA	1.6982
P06454	Prothymosin alpha [Cleaved into: Prothymosin alpha, N-terminally processed; Thymosin alpha-1]	PTMA	2.9158
P06703	Protein S100-A6	S100A6	1.3120
P06733	Alpha-enolase	ENO1	1.6095
P06753	Tropomyosin alpha-3 chain	TPM3	1.5452
P07195	L-lactate dehydrogenase B chain	LDHB	1.7715
P07737	Profilin-1	PFN1	1.1463
P07858	Cathepsin B	CTSB	1.0506
P07900	Heat shock protein HSP 90-alpha	HSP90AA1	1.0298
P09382	Galectin-1	LGALS1	0.9991
P10599	Thioredoxin	TXN	1.4176
P11021	78 kDa glucose-regulated protein	HSPA5	-1.8644
P13646	Keratin, type I cytoskeletal 13	KRT13	-1.1805
P13647	Keratin, type II cytoskeletal 5	KRT5	-2.0880
P13667	Protein disulfide-isomerase A4	PDIA4	-1.1685
P14618	Pyruvate kinase PKM	PKM	1.2771
P14625	Endoplasmin	HSP90B1	-1.1513
P14923	Junction plakoglobin	JUP	-2.1725
P15531	Nucleoside diphosphate kinase A	NME1	1.0309
P17066	Heat shock 70 kDa protein 6	HSPA6	-0.9634
P21926	CD9 antigen	CD9	-1.1057
P23528	Cofilin-1	CFL1	0.9401
P25789	Proteasome subunit alpha type-4	PSMA4	1.3997
P26038	Moesin	MSN	0.9740
P26641	Elongation factor 1-gamma	EEF1G	0.9392
P29401	Transketolase	TKT	1.1985
P30464	HLA class I histocompatibility antigen, B-15 alpha chain	HLA-B	1.0711
P31946	14-3-3 protein beta/alpha	YWHAB	1.1415
P37802	Transgelin-2	TAGLN2	1.5141
P39687	Acidic leucine-rich nuclear phosphoprotein 32 family member A	ANP32A	0.9083
P51659	Peroxisomal multifunctional enzyme type 2	HSD17B4	-0.8931
P51858	Hepatoma-derived growth factor	HDGF	1.2002
P52209	6-phosphogluconate dehydrogenase, decarboxylating	PGD	1.3715
P55854	Small ubiquitin-related modifier 3	SUMO3	0.9421
P58107	Epiplakin	EPPK1	-1.5691
P60174	Triosephosphateisomerase	TPI1	1.3449
P61981	14-3-3 protein gamma	YWHAG	0.8865
P62258	14-3-3 protein epsilon	YWHAE	1.4509
P62263	40S ribosomal protein S14	RPS14	0.8956
P62753	40S ribosomal protein S6	RPS6	1.0464
P62857	40S ribosomal protein S28	RPS28	1.1781
P62873	Guanine nucleotide-binding protein G(I)/G(S)/G(T) subunit beta-1	GNB1	-0.8858
P63104	14-3-3 protein zeta/delta	YWHAZ	1.3496
P63244	Receptor of activated protein C kinase 1	GNB2L1	1.0701
P67936	Tropomyosin alpha-4 chain	TPM4	1.1676
P80723	Brain acid soluble protein 1	BASP1	1.1421
Q01105	Protein SET	SET	1.6510
Q03252	Lamin-B2	LMNB2	-1.0721
Q04695	Keratin, type I cytoskeletal 17	KRT17	-1.3556
Q13045	Protein flightless-1 homolog	FLII	-1.1347
Q13185	Chromobox protein homolog 3	CBX3	1.2688
Q15084	Protein disulfide-isomerase A6	PDIA6	-0.9692
Q16891	MICOS complex subunit MIC60	IMMT	-0.9737
Q7KZF4	Staphylococcal nuclease domain-containing protein 1	SND1	-3.5940
Q7Z6K1	THAP domain-containing protein 5	THAP5	-5.1194
Q99729	Heterogeneous nuclear ribonucleoprotein A/B	HNRNPAB	-0.9374
Q9Y4L1	Hypoxia up-regulated protein 1	HYOU1	-1.3786

### 3.3 Gene ontology analysis of Proteins Regulated by ANXA5 in NCl-H520 Cells

**Fig 2:**
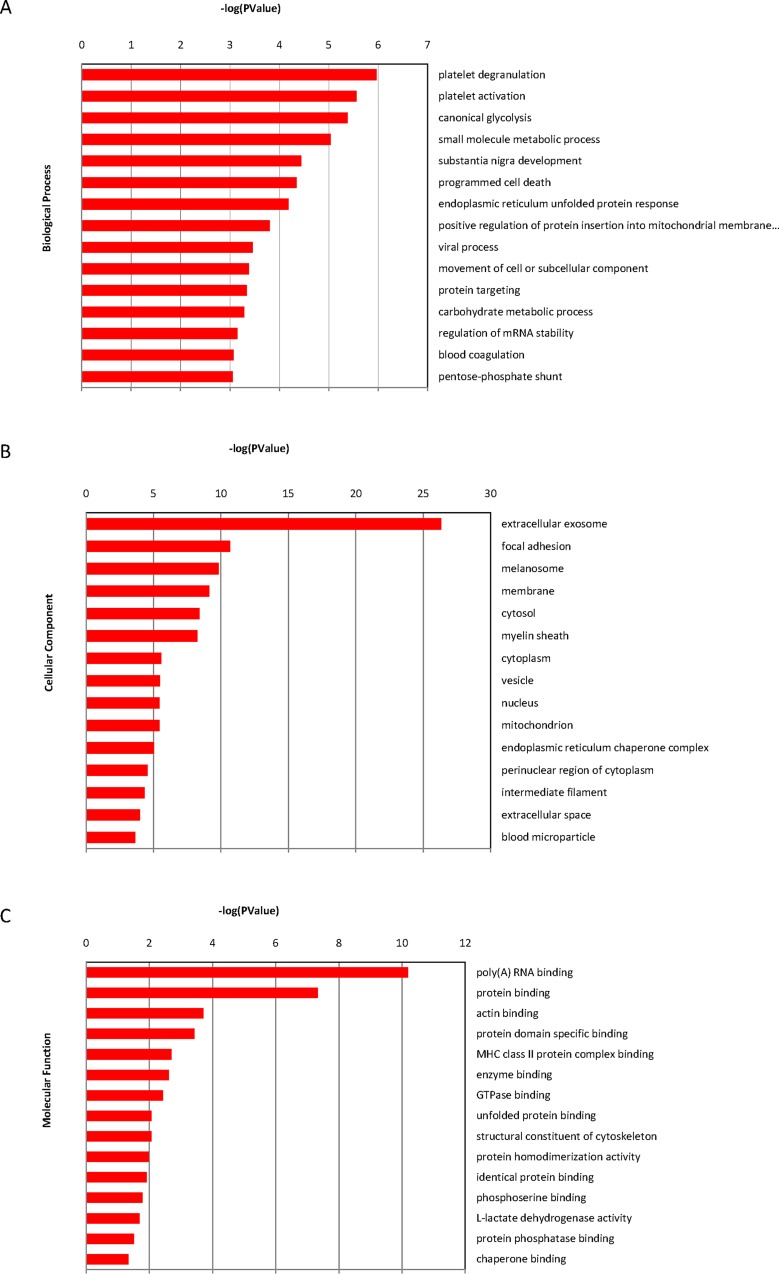
The differentially expressed proteins as analyzed by GO terms. (A) cellular component, molecular function (B) and biological process (C).

To better understand the biological pathways being regulated by ANXA5, the selected 70 proteins were annotated into several functional categories with DAVID. The majority of the differentially regulated proteins are involved in platelet degranulation activity, followed by platelet activation, canonical glycolysis activity, small molecule metabolic process, substantia nigra development and programmed cell death (Fig 2A). Furthermore, the identified annotated proteins were localized in different sub-cellular compartments and functions, namely extracellular exosome, focal adhesion, melanosome, membrane, cytosol and myelin sheath (Fig 2B). In addition, GO biological process annotation analysis revealed that the molecular events pertinent to the functioning of multi-catabolic, metabolic and biosynthetic process, including poly(A) RNA binding, protein binding, actin binding, enzyme binding, and protein phosphatase binding (Fig 2C).

### 3.4 Pathway analysis by IPA software

**Fig 3:**
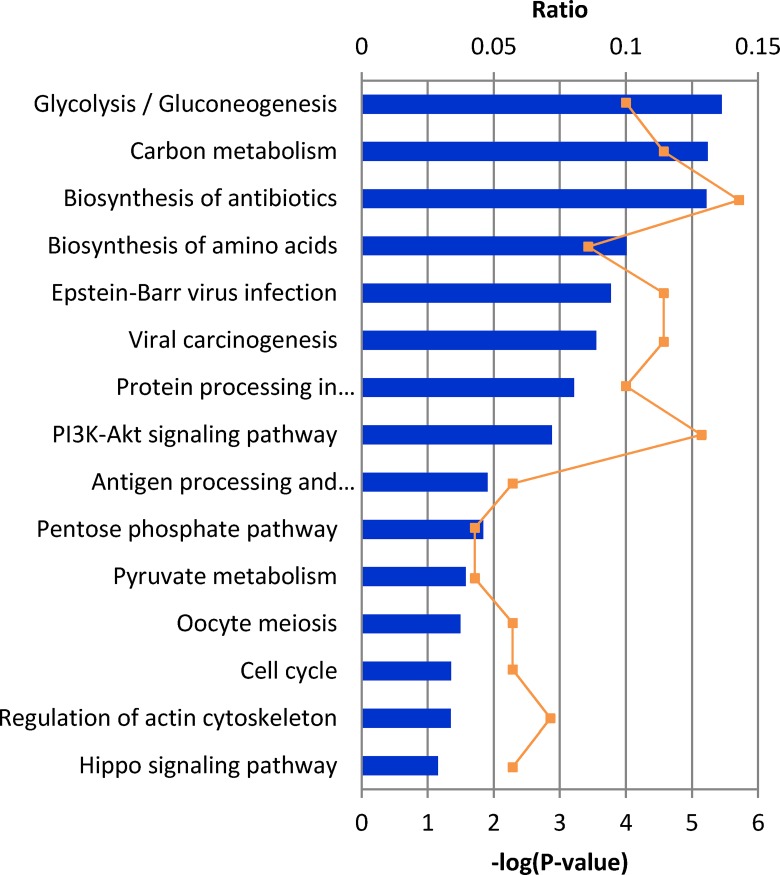
IPA analysis of proteins changing in abundance after ANXA5 transfection. Association of canonical signaling pathways with modulated proteins are shown. The proteins which demonstrated significant change (95% confidence interval with statistical significance) were subjected to IPA analysis. The top 15 canonical pathways were identified as significantly altered upon ANXA5 modulation. The line bar represents the threshold of significance (p = 0.05).

The relationships, canonical pathways and interaction networks analysis of the differentially expressed proteins were further interpreted using Ingenuity Pathway Analysis (IPA, Ingenuity Inc., Mountain View, CA; http://www.ingenuity.com), which is based entirely on findings reported in the literature. In total, 15 canonical pathways were identified from the differentially expressed proteins. Among these, the most significant canonical pathways were glycolysis and gluconeogenesis, when ranked by significance (p-value < 2 × 10^−5^) as well as by ratio (1.2E-01). Further, as shown in Fig 3, an involvement of several important signaling pathways (carbon metabolism, biosynthesis of amino acids, protein processing in endoplasmic reticulum) in the biological association of ANXA5 was identified.

**Fig 4:**
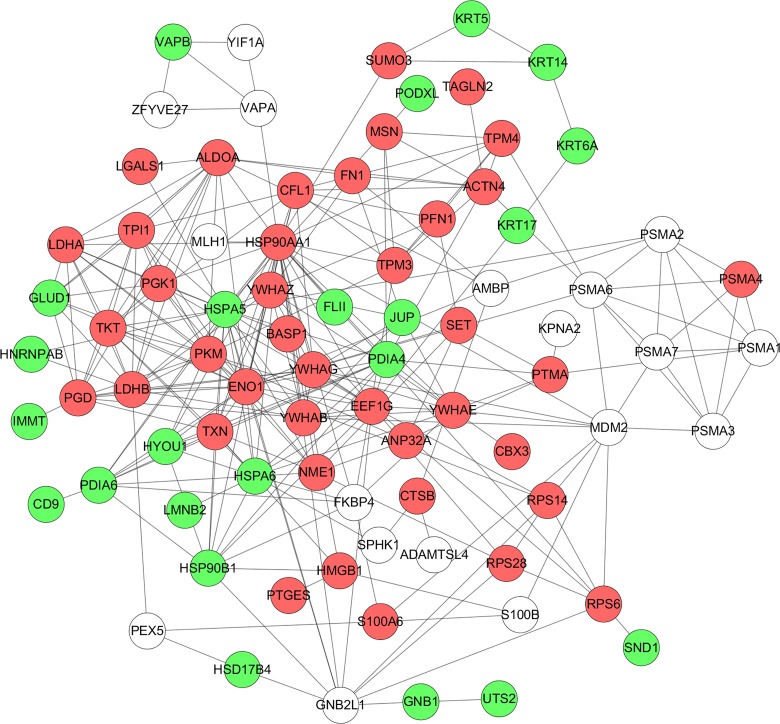
Protein-protein interaction by IPA analysis. IPA was further used to determine the protein-protein interactions among modulated proteins. The upregulated proteins upon ANXA5 modulation are represented in red color whereas the downregulated proteins are shown in green. The un-colored nodes indicate additional proteins of this network that were not spotted by the proteomics analysis.

The protein-protein networks of ANXA5 regulated proteins were algorithmically generated based on their connectivity. The biggest network involves 64 differentially expressed proteins, which can link together into one network through direct interaction or only one intermediate partner. Multiple central nodes, namely HSPA5, FN1, PDIA6, ENO1, ALDOA, JUP, HSP90AA1 and KRT6A, were identified from protein-protein networks (Fig 4).

### 3.5 Confirmation of Differentially Expressed Proteins by Western Blotting

**Fig 5:**
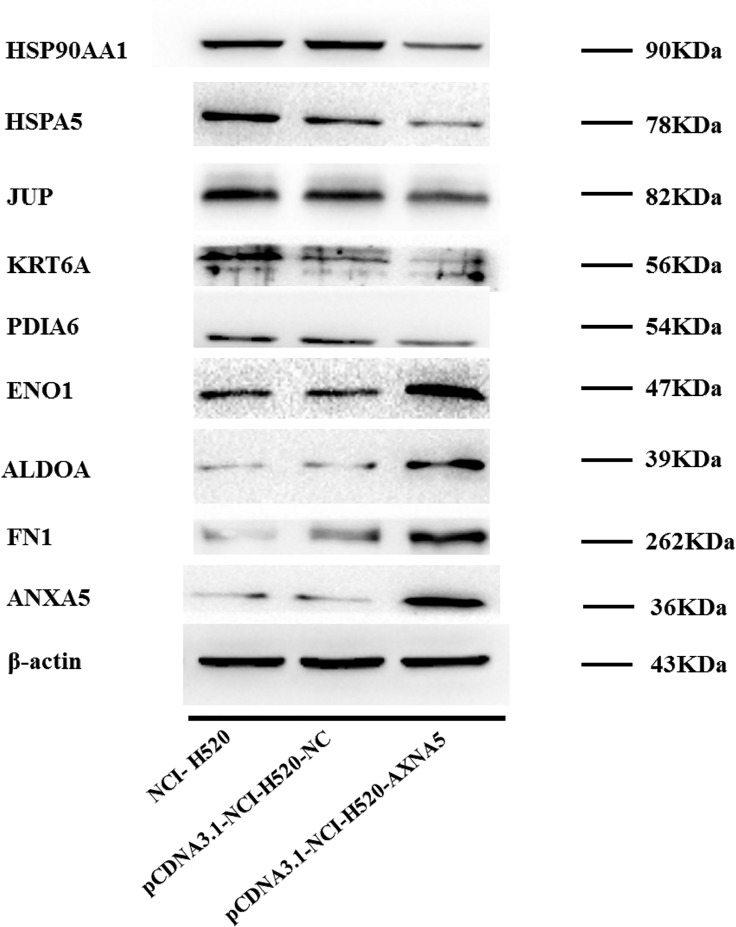
Validation of differentially expressed proteins by immunoblot analysis.

The computational network analysis was applied to capture the complex and dynamic nature of the changes in the H520 cell upon ANXA5 modulation. The structure of the network generated from the proteomic analysis could identify several proteins as central hubs of connectivity and highlight the complex interactions among its various nodes. Thus, the expression of some central hubs identified in the ANXA5 modulated protein network were chosen to be validated in control and ANXA5 transfected H520 cells through western blotting assay. Consistent with the proteomics results, PDIA6, HSPA5, JUP and KRT6A were found to be down-regulated, whereas FN1, ENO1 and ALDOA were found to be up-regulated in ANXA5 transfected H520 cells (Fig 5). In addition, one of the central hubs, Hsp90 alpha was found to be down-regulated, which was inconsistent with the proteomics results.

## 4. Discussion

The deregulation of ANXA5 was observed as a potential marker in a range of tumors [3]. However, the associations of ANXA5 with lung cancer progression, invasion and metastasis are very complex and remains poorly understood. This study was undertaken to identify the alterations in the lung squamous carcinoma cell proteome caused by ANXA5 transfection. Our previous results from crystal violet staining, CCK-8 assay, scratch wound assay, and Transwell assay proved that upregulation of ANXA5 may inhibit the proliferation, migration, and invasion abilities of NCI-H520 cells in vitro[6]. And the ANXA5 transfected H520 cells might be served as an important and ideal cellular model to investigate the mechanism of ANXA5 in lung cancer.

In this study, we performed a comparative proteomic analysis to profile differentially expressed proteins modulated by ANXA5. As described in‘Results’, our proteomics analysis have revealed that ANXA5 modulates multiple key pathways, which are relevant to cancer cell growth and metabolism. It is worth noting that a big proportion of the proteins affected by ANXA5 are involved in cellular catabolism and metabolism, which is a key step in tumorigenesis and tumor progression. There is increasing evidence that cells undergo metabolic reprogramming to fulfill their energy requirement [32–34], which has been suggested as a key hallmark of cancer progression.

Based on IPA analysis (Fig 3), glycolysis and gluconeogenesis protein were identified as the top canonical pathway modulated by ANXA5 in our study. The proteins identified by IPA analysis that are directly involved in glycolysis and gluconeogenesis pathways include ALDOA, ENO1 and TPI1. Glycolysis has been proposed as a main source of ATP for tumor cell survival upon detachment and during migration [34, 35]. The increased glycolytic activity induced by ANXA5 in H520 cell suggests that lung squamous cells benefit from up-regulating glycolysis in response to the inhibition on the proliferation, migration and invasion capabilities modulated by ANXA5.

Further, multiple central nodes (FN1, ENO1, HSPA5, Hsp90alfa and JUP) were identified in IPA analysis to be modulated by ANXA5 (Fig 4). These central nodes are very interesting and relevant to cancer development and progression. Fibronectin 1 (FN1) is a glycoprotein that is involved in cell adhesion and migration processes including embryogenesis, wound healing, blood coagulation, host defenses and metastasis. FN1 has been suggested as a potential biomarker in various cancers [36, 37]. In lung cancer, fibronectin can promote lung cancer cell migration by activating FAK signaling [38, 39]. Our proteomics data shows the remarkablely increased expression of FN1 (Table 2, Fig 5). Enolase 1 (ENO1) is a glycolytic enzyme responsible for the ATP-generated conversion of 2-phosphoglycerate to phosphoenolpyruvate, known as endoplasmic reticulum (ER) resident chaperon, and has multifunctional responses. Overexpression of ENO1 is associated with tumor progression and invasion [40, 41]. ENO1 has been suggested as a biomarker candidate for lung cancer [42]. In this study, we found increased level of ENO1 at the proteomics level in H520 cells (Table 2, Fig 5).

In this study, we found a significant decrease in two heat shock family members, Heat shock protein A5 (HSPA5) (Table 2, Fig 5) and Heat shock protein 90 (Hsp90) (Fig 5). HSPA5 also known as binding immunoglobulin protein (Bip) or glucose regulated protein 78 (Grp78), belongs to the heat shock protein 70 kDa family. Hsp90 is known as an ATPase-dependent molecular chaperone, ubiquitously expressed in eukaryotic cells. HSPA5 [43, 44] and Hsp90 [45, 46] play an indispensable role in normal cellular homeostasis by regulating the folding, stability, function of client proteins including protein kinases and transcription factors, many of which are important for the proliferation and survival of cancer cells. However, there is no reported role for HSPA5 in lung cancer at present, while Hsp90 has been regarded as serum biomarker [47] and therapeutic target [45, 48] in lung cancer.

Interestingly, in our study, we found that ANXA5 also resulted in a marked decrease of four members of the Keratin family, KRT5, KRT6A, KRT14 and KRT17 (Table 2, Fig 5). The Keratin family belongs to intermediate filament proteins, predominantly expressed in epithelial cells, which play an essential role in keeping cell structure integrity and stability[49], cell mobility and proliferation [50, 51] and signaling [52]. Prognostic associations of KRT5 [53–55] and KRT14 [53, 54] have been implicated in lung cancer, while the relationship and molecular mechanisms of KRT6A and KRT17 in the lung cancer are still unknown and should be further elucidated.

## 5. Conclusion

In summary, our quantitative proteomics approach identified ANXA5 as a pleotropic regulator in the lung cancer cells. We provided evidence that up-regulation of ANXA5 activated the glycolysis and gluconeogenesis of tumor cells. Some ANXA5-regulated proteins such as FN1, ENO1, heat shock and Keratin family numbers are closely related to invasion and progression of lung cancer. These findings extend our understanding of the underlying molecular mechanism of ANXA5 in lung cancer.

## Supporting Information

S1 Table The detailed information of all quantified proteins. (XLSX)
